# Multilocus Sequence Typing of *Mycoplasma pneumoniae*, Japan, 2002–2016

**DOI:** 10.3201/eid2410.171194

**Published:** 2018-10

**Authors:** Mariko Ando, Miyuki Morozumi, Yoko Adachi, Kimiko Ubukata, Satoshi Iwata

**Affiliations:** Keio University School of Medicine, Tokyo, Japan (M. Ando, M. Morozumi, Y. Adachi, K. Ubukata, S. Iwata);; National Cancer Center Hospital, Tokyo (S. Iwata)

**Keywords:** *Mycoplasma pneumoniae*, multilocus sequence typing, MLST, macrolide-resistant *Mycoplasma pneumoniae*, macrolide-sensitive *Mycoplasma pneumoniae*, bacteria, Japan

## Abstract

In Japan, *Mycoplasma pneumonia*e resistance to macrolides is high. To compare sequence types (STs) of susceptible and resistant isolates, we performed multilocus sequence typing for 417 isolates obtained in Japan during 2002–2016. The most prevalent ST overall was ST3, for macrolide-resistant was ST19, and for macrolide-susceptible were ST14 and ST7.

Macrolide-resistant *Mycoplasma pneumonia*e was first isolated in Japan in 2000 (*1*). Macrolide-resistant *M. pneumoniae* is highly prevalent in Asia and has been reported from several parts of the world (*2**–**7*). During a 2011–2012 outbreak in Japan, the resistance rate was as high as 90% (*8*). Molecular typing methods have been developed for *M. pneumoniae* and include multilocus variable-number tandem-repeat analysis (MLVA), P1 typing, and others (*9*). In recent years, multilocus sequence typing (MLST) involving molecular analysis of 7–8 housekeeping genes has been applied to various bacterial pathogens (*10**,**11*). In 2014, MLST for *M. pneumoniae* was devised by Brown et al. (*12*). Using this MLST method for *M. pneumoniae*, we compared sequence types (STs) of macrolide-susceptible and macrolide-resistant *M. pneumoniae* isolates from Japan and examined their evolutionary relationships. As is commonly performed for this pathogen, we also typed the P1 adhesin gene.

## The Study

Using the random function of Excel 2010 (Microsoft, Redmond, CA, USA), we randomly selected 417 *M. pneumoniae* isolates (372 from children, 45 from adults) from 1,084 isolates obtained from patients throughout Japan who had had pneumonia or bronchitis during 2002–2016. Samples were not collected in 2014. The strains were isolated from clinical samples sent from 31 medical institutions in Japan to the Department of Infectious Diseases at Keio University School of Medicine in Tokyo. Of these strains, 232 (55.6%) were macrolide-susceptible *M. pneumoniae* and 185 (44.4%) were macrolide-resistant *M. pneumoniae* that had 23S rRNA point mutations. According to *M. pneumoniae* numbering, these mutations were A2063G (n = 163), A2063T (n = 10), A2064G (n = 10), A2063C (n = 1), or C2617A (n = 1). We used microdilution methods with pleuropneumonia-like organism broth (Difco, Detroit, MI, USA) to determine MICs for 6 antimicrobial agents against these strains of macrolide-susceptible and macrolide-resistant *M. pneumoniae* (Table 1). 

**Table 1:** Antimicrobial activity of 6 antimicrobial agents against 417 *Mycoplasma pneumoniae* strains, Japan, 2002–2016*

Antimicrobial agent and *M. pneumoniae* macrolide-resistance level	MIC, μg/mL†		
50%	90%	Range
Clarithromycin			
Susceptible	0.0039	0.0078	0.00195 to 0.031
Resistant	64	>64	0.5 to >64
Azithromycin			
Susceptible	0.00049	0.00098	0.00012 to 0.00195
Resistant	64	64	0.031 to >64
Minocycline			
Susceptible	0.5	1	0.031 to 1
Resistant	0.25	1	0.063 to 1
Doxycycline			
Susceptible	0.5	0.5	0.063 to 0.5
Resistant	0.25	0.5	0.125 to 0.5
Levofloxacin			
Susceptible	0.5	1	0.125 to 1
Resistant	0.5	1	0.5 to 1
Tosufloxacin			
Susceptible	0.5	0.5	0.25 to 1
Resistant	0.5	0.5	0.25 to 2

*Macrolide-susceptible *M. pneumoniae* = 232; macrolide-resistant *M. pneumoniae* = 185. †MIC ranges for macrolides were 16 to >64 μg/mL for A2063G (n = 163) and A2064G (n = 10) and >64 μg/mL for A2063C (n = 1). MIC for azithromycin was affected by the mutations A2063T (1–4 μg/mL) (n = 10) and C2617A (0.031 μg/mL) (n = 1). MIC for clarithromycin was affected by C2617A (0.5 μg/mL). MICs for quinolone and tetracycline in both macrolide-susceptible and macrolide-resistant *M. pneumoniae* were almost the same.

For MLST analysis, we sequenced 8 housekeeping genes (*ppa*, *pgm*, *gyrB*, *gmk*, *glyA*, *atpA*, *arcC*, and *adk*) in each strain by using the 8 primer sets described in the MLST database (https://pubmlst.org/mpneumoniae/) (*12*). New alleles and STs were registered in the *M. pneumoniae* MLST database. To determine relationships between STs, we conducted clonal complex (CC) analysis by using eBURST version 3.1 (http://eburst.mlst.net/v3/mlst_datasets/). Frequency analysis was performed with the Fisher exact test.

Typing of the P1 adhesin gene in *M. pneumoniae* was performed as previously described (*13*). After PCR, the purified DNA products were treated with a restriction enzyme (*Hae*III) for 1 hour. The standard stains M129 (ATCC 29342) and FH (ATCC 15531) were used as controls.

**Figure 1:**
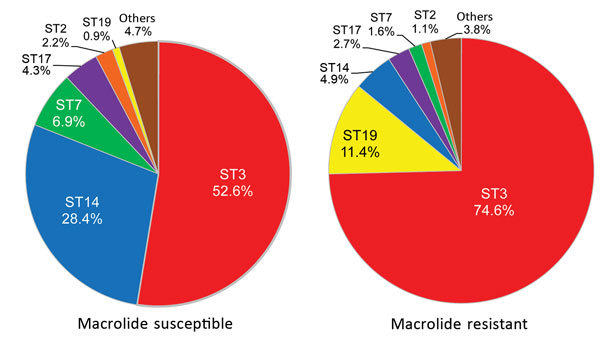
Multilocus sequence typing results for 232 macrolide-susceptible and 185 macrolide-resistant *Mycoplasma pneumoniae* isolates, Japan, 2002–2016. Ten STs were identified for macrolide-susceptible and 12 STs for macrolide-resistant *M. pneumoniae*. ST, sequence type.

Among macrolide-susceptible *M. pneumoniae* isolates, the most prevalent STs were ST3 (52.6%), ST14 (28.4%), and ST7 (6.9%) (Figure 1). Among macrolide-resistant isolates, the most prevalent ST was also ST3 (74.6%), but the next most prevalent were ST19 (11.4%) and ST14 (4.9%). ST7 was more prevalent in macrolide-susceptible than in macrolide-resistant isolates (p = 0.008); ST19 was more prevalent in macrolide-resistant isolates (p<0.001). Although ST14 was prevalent among macrolide-susceptible *M. pneumoniae*, it also was represented among macrolide-resistant *M. pneumoniae*, associated with the 23S rRNA mutation A2064G. We did not identify any differences in ST frequencies between isolates from children and adults or between areas in Japan.

When we compared the relationship between year of strain isolation and ST (Table 2), ST3 accounted for most macrolide-susceptible *M. pneumoniae*, but ST14 also was present in every year; in other words, 2 STs were consistently common among macrolide-susceptible *M. pneumoniae*. In 2016, infection with macrolide-susceptible *M. pneumoniae,* especially ST7 and ST14, was epidemic. Among macrolide-resistant isolates, most were ST3. Although ST3 was identified more frequently in macrolide-susceptible isolates during 2002–2006, its identification in macrolide-resistant isolates has increased rapidly since then, and macrolide-resistant ST3 became more common in 2008. ST19 has been identified since the *M. pneumoniae* epidemic in 2006.

**Table 2:** Relationship between year isolated and ST among 232 macrolide-susceptible and 185 macrolide-resistant *Mycoplasma pneumoniae*, Japan, 2002–2016*

Year	Macrolide-susceptible *M. pneumoniae*								Subtotal	Macrolide-resistant *M.* *pneumoniae*								Subtotal	Total
	CC1				CC2			Other ST		CC1				CC2			Other ST		
	ST 3	ST 17	ST 19		ST 2	ST 7	ST 14			ST 3	ST 17	ST 19		ST 2	ST 7	ST 14			
2002	8				3	3	10	3	27							2		2	29
2003	13					3	2	1	19	3				2				5	24
2004	13						2	2	17	4						1		5	22
2005	12					1	3		16	3							1	4	20
2006	24	2			1		3	2	32	15		5					2	22	54
2007	10	4			1		8		23	12		7						19	42
2008	7	1					7		15	15	2					1		18	33
2009	8						2		10	12		1						13	23
2010	4		1				2	3	10	8		4					1	13	23
2011	5	1					1		7	13		2				3	1	19	26
2012	8		1				2		11	16	3	2						21	32
2013	3	1							4							1		1	5
2015	2					1	5		8	25								25	33
2016	5	1				8	19		33	12					3	1	2	18	51
Total	122	10	2		5	16	66	11	232	138	5	21		2	3	9	7	185	417
%	52.6	4.3	0.9		2.2	6.9	28.4	4.7	100	74.6	2.7	11.4		1.1	1.6	4.9	3.8	100	

*CC, clonal complex; ST, sequence type.

**Figure 2:**
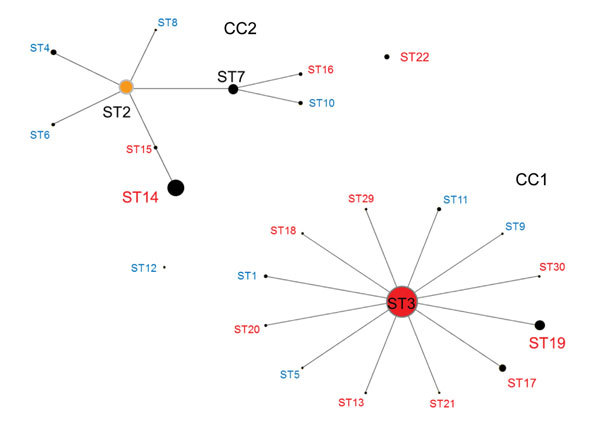
Relationship between CC and ST for *Mycoplasma pneumoniae* isolates by eBURST version 3.1 analysis (http://eburst.mlst.net/v3/mlst_datasets/). Data included 417 strains from Japan, 2002–2016, and 62 strains isolated from the United Kingdom, the United States, China, and France. For all isolates, 24 STs were identified. The size of each circle is proportional to the number of isolates for each ST. Red indicates isolates detected in Japan only; blue indicates isolates detected in the United Kingdom, the United States, China, and France but not Japan; black indicates isolates detected in all 5 countries. CC, clonal complex; ST, sequence type.

Data showing the relationship between CC and STs according to eBURST (Figure 2) include the 417 strains in this study and 62 strains registered to the MLST database from other countries. Of the 2 CC clusters, the center of CC1 was ST3, from which new STs including ST19 and ST17 arose with >1 alleles mutated. CC1 appears more frequently in macrolide-resistant *M. pneumoniae* (p<0.001). The center of CC2 was ST2, and almost all strains in Japan belonged to ST7 and ST14. ST22 did not belong to either CC1 or CC2, but 5 of 8 of its alleles were identical to those of CC1. In certain areas of Japan, ST22 has emerged in macrolide-resistant and macrolide-susceptible *M. pneumoniae*.

P1 typing analysis indicated that *M. pneumoniae* STs belonging to CC1 and ST22 were type 1, and STs belong to CC2 were type 2. ST14 and ST15 were type 2a, which is a subtype of P1 type 2.

## Conclusions

Molecular analysis by MLST is conducted commonly for various bacterial pathogens worldwide but not for *M. pneumoniae.* We identified differences in STs between macrolide-susceptible and macrolide-resistant *M. pneumoniae* in Japan. Of the STs noted among isolates from 4 countries (the United Kingdom, the United States, China, and France), ST3 and ST14 were identified often in Japan, but other STs were not identified in Japan (Figure 2). During 1977–2011, the most prevalent *M. pneumoniae* STs in England and Wales were ST2, ST3, ST4, and ST11 (*14*). In this respect, STs reported from Japan show patterns quite different from those from other countries. The most prevalent STs in Japan were ST3, ST14, ST19, and ST7. In particular, ST3 of macrolide-susceptible *M. pneumoniae* was the most prevalent during 2002–2006, the early period of our surveillance, but ST3 of macrolide-resistant *M. pneumoniae*, with a point mutation of A2063G in the 23S rRNA gene, increased rapidly since an outbreak of *M. pneumoniae* infection in 2006, and macrolide-resistant *M. pneumoniae* became most common after 2008. This change toward resistance occurred in parallel with increased use of macrolides in children. ST14 and ST7 were more prevalent among macrolide-susceptible than macrolide-resistant *M. pneumoniae*; ST19 was prevalent among macrolide-resistant *M. pneumoniae*.

We detected 2 types of the P1 adhesin gene, types 1 and 2 (which includes 2a), and analyzed them in terms of STs. Recent studies have identified various subtypes through comprehensive genomic comparisons (*15*). Similar to the results of MLST, P1 gene types have shown diversification. We did not conduct MLVA in this study, but a previous study found CC1 and CC2 to differ in MLVA type: CC1 contained MLVA type 4572, and CC2 contained MLVA types 3662 and 3562 (*12*).

In summary, macrolide-resistant *M. pneumoniae* are emerging and increasing. Surveillance of macrolide-resistant *M. pneumoniae* based on molecular epidemiology, including STs and P1 adhesion gene identifications, are necessary to clarify worldwide trends and address public health concerns.
